# Characterization of Whey Protein Isolate–Soymilk Complexes Modified by Transglutaminase and Their Application inYuba Film

**DOI:** 10.3390/foods14162916

**Published:** 2025-08-21

**Authors:** Junliang Chen, Yao Chen, Weiwei Cao, Tongxiang Yang, Linlin Li, Wenchao Liu, Xu Duan, Guangyue Ren

**Affiliations:** 1 College of Food and Bioengineering, Henan University of Science and Technology, Luoyang 471023, China; chenjl2020@haust.edu.cn (J.C.); 15039287183@163.com (Y.C.); txyamy@163.com (T.Y.); linlinli2020@126.com (L.L.); wen_chaoliu@163.com (W.L.); duanxu_dx@163.com (X.D.); guangyueyao@163.com (G.R.); 2 Henan Province Engineering Research Center of Agricultural Products Processing Equipment, Luoyang 471000, China; 3 Henan Province Engineering Technology Research Center of Agricultural Product Drying Equipment, Luoyang 471000, China

**Keywords:** transglutaminase, yuba, soymilk, whey isolate protein

## Abstract

Transglutaminase (TGase) improves protein structure by facilitating cross-linking reactions. However, the effects of TGase on the physicochemical properties of whey protein isolate (WPI)–soymilk complexes and their applications in yuba remain unclear. Therefore, the impacts of TGase concentration on the free sulfhydryl content, free amino content, particle size, and structure of WPI–soymilk complexes and their film-forming properties were studied. The results showed that the physicochemical properties of the composite soymilk were changed by the TGase-induced cross-linking reaction of protein. Compared with the composite soymilk without TGase modification, the particle size of the WPI–soymilk complexes increased from 707.99 ± 9.47 nm to 914.41 ± 2.8 nm as the TGase concentration increased, and the complexes remained relatively stable at low TGase concentrations. TGase modification changed the tertiary structure of the WPI–soymilk complexes. The composite yuba with 0.01% and 0.03% levels of TGase had a higher β-sheet content than composite yuba without addition of TGase. The surface hydrophobicity of composite soymilk was decreased by all the addition levels of TGase. Meanwhile, the TGase-modified composite protein with 0.03% TGase had the lowest free sulfhydryl (35.92 μg/g) and amino groups (0.46). Additionally, the tensile strength of the composite yuba with 0.05% TGase addition reached a peak of 1.66 ± 0.02 MPa, which was 7.8% higher than that of the composite yuba without TGase addition. The SEM results revealed that the composite yuba with 0.01–0.03% TGase addition exhibited a dense and non-porous film structure. Moreover, all the composite yuba with TGase addition had a reduced rate of yuba cooking loss. This study contributes to enhancing the yield and mechanical properties of traditional yuba.

## 1. Introduction

Yuba, with its distinctive flavor and high nutrition value, is popular in East Asia and is obtained by forming a film on the surface of heated soymilk. Recent studies demonstrated that the combination of plant and animal proteins could generate synergistic effects [1]. Our earlier work on composite yuba using whey protein isolate showed noticeable improvements in mechanical strength compared to traditional yuba. The heat-induced aggregation of proteins and their adsorption behavior at the liquid/gas interface significantly influence the product conformation during yuba formation. Cai et al. demonstrated that the protein aggregate concentration at the interface played a key role in driving nonlinear growth in film thickness [2]. Researchers commonly employ various pretreatment methods for changing protein quality, including physical treatments, chemical modifications, biological modifications, and complexes of proteins and other additives. Enzymatic cross-linking has received more attention than other methods due to its non-toxic nature and high consumer acceptance, and has the potential to further improve the mechanical properties of composite yuba.

Transglutaminase (TGase) is a commercially available food-grade enzyme widely utilized to modify protein function via covalent cross-linking. The approachability of lysine residues and glutamine within protein could influence TGase activity, which facilitates deamidation and cross-linking reactions. Cross-linking occurs via the reaction of acyl transfer between the γ-carboxamide groups and lysine ε-amino groups or other primary amines. As an acyl acceptor, water in the absence of primary amines leads to TGase-mediated hydrolysis of glutamine γ-carboxamide groups and consequent deamidation [3]. Furthermore, TGase was demonstrated to significantly improve the mechanical strength of protein-based films [4]. Jin et al. employed TGase to improve the cross-linking of plant protein, which produced tofu with a softer texture and lower cutting stress [5]. Wu et al. reported that TGase catalyzed interfacial cross-linking of rice bran proteins and increased the interfacial elastic modulus [6]. However, the compact structure of proteins often buries active sites within the molecular interior, which limits reactive group exposure and results in low catalytic efficiency for cross-linking reactions. Consequently, pretreatment methods may promote structural unfolding and surface residue exposure, thereby enhancing enzymatic reactions.

As an efficient and eco-friendly physical technology, ultrasound demonstrates many advantages in improving protein function. During sonication, pressure fluctuations generated by acoustic wave propagation through aqueous media lead to the implosive collapse and formation of microbubbles in liquids. The intense hydrodynamic shear forces, pressure, and temperature induce both physical and chemical modifications in food materials [7]. Sonication technology enhances protein functionality through structure modification and exposure of active sites [8]. Therefore, ultrasonic pretreatment is a promising approach for creating recognizable sites to facilitate enzymatic cross-linking and high-quality products. For example, Arzeni et al. demonstrated that ultrasonic processing reduced the particle size of soybean protein isolate (SPI) and promoted TGase-induced SPI gel formation [9]. Ma et al. revealed that ultrasound and enzyme treatment altered the structure of SPI [10], which improved the water-holding capacity (WHC) and gel strength of the SPI gel. Thus, we hypothesized that TGase could modulate the film-forming behavior of ultrasound-treated composite soymilk. Therefore, effects of TGase concentration on the particle size, polydispersity index (PDI), intrinsic fluorescence spectra, free amino groups, free sulfhydryl, and surface hydrophobicity content of ultrasound-treated composite soymilk were studied. Furthermore, the influence of TGase on the quality, including yield, mechanical properties, and cooking loss ratio, of yuba produced by TGase-modified WPI–soymilk complexes was evaluated.

The objective of this study is to reveal the regulation of the physicochemical properties of WPI–soymilk complexes modified by transglutaminase and yuba film by TGase.

## 2. Materials and Methods

### 2.1. Materials

WPI was ordered from Milk Specialties Global (Eden Prairie, MN, USA). Soybean was obtained from Dazhang supermarket (Luoyang, China). TGase was purchased from Shanghai Qingrui Food Technology Co., Ltd. (Shanghai, China). Reagents of analytical grade were utilized for all the experiments.

### 2.2. Preparation of Ultrasound-Treated Composite Soymilk and Yuba Films Modified by TGase

Soybean (100 g) soaked at 20 °C in 400 mL of deionized water for 12 h was ground with a soybean-to-water ratio of 1:10 (*w*/*v*). The above slurry was filtered to obtain soymilk. Based on preliminary experiments, 12 g of WPI was added to the filtered soymilk and homogeneously mixed with a magnetic stirrer for 30 min to obtain composite soymilk.

The above mixed soymilk (500 mL) was treated with 200 W of ultrasonic power for 15 min with a pulse duration of 2 s off and 3 s on. After ultrasonic treatment, varying concentrations of 110 U/g TGase (0.01%, 0.03%, 0.05%, 0.07%, and 0.09%) were supplemented to the composite soymilk at 50 °C for 30 min based on the results of preliminary experiments. The composite soymilk was then boiled to inactivate the TGase, and it was placed in a container in an 85 °C water bath for film formation. The air–water interface could form yuba film, and the films were collected every 15 min. The above films were dried at 60 °C for 6 h to obtain yuba. The method flow of producing composite yuba was shown in Supplementary Materials picture. The composite soymilk with TGase addition concentrations of 0, 0.01%, 0.03%, 0.05%, 0.07%, and 0.09% was labeled as US-TG-0 (Control), US-TG-0.01%, US-TG-0.03%, US-TG-0.05%, US-TG-0.07%, and US-TG-0.09%, respectively.

### 2.3. Characterization of Modified Composite Soymilk

#### 2.3.1. Particle Size Distribution (PSD) and Particle Size

Before determining the PSD and particle size of the composite soymilk, distilled water was adopted to prepare a 200× dilution of the composite soymilk. The particle size and PSD were measured by a BeNano 90Zeta nanoparticle size potentiostat (Bettersize, Dandong, China).

#### 2.3.2. Surface Hydrophobicity

A 10 mM phosphate buffer (pH 7.0) was adopted to prepare 0.01–0.2 mg protein/mL soymilk, and 20 μL of 8.0 mM ANS agent was added into the soymilk. An Agilent Cary eclipse spectrofluorometer (Agilent Technologies Inc., Santa Clara, CA, USA) was adopted to measure the fluorescence intensity of soymilk. The excitation wavelength was set at 390 nm, and the scanning wavelength ranged from 400 nm to 600 nm.

#### 2.3.3. Free Sulfhydryl (SH) Content

The free SH content in composite soymilk was determined according to the method reported by Kang et al. [11]. A Tris-Gly solution (2.8 mL) was mixed with the sample (0.2 mL), followed by the addition of 20 μL of 4 mg/mL DTNB. The mixture was centrifuged at 10,000× *g* for 5 min. The absorbance of the supernatant at 412 nm was determined by a microplate reader.

#### 2.3.4. Intrinsic Fluorescence Spectrum Measurement

A Cary eclipse spectrofluorometer (Agilent, Agilent Technologies Inc., Santa Clara, CA, USA) was used to determine the intrinsic fluorescence spectra of the composite soymilk following the method reported by Cheng et al. [12]. The composite soymilk was diluted at a ratio of 1:200 before determination. Fluorescence measurements were performed with an excitation wavelength of 280 nm, and emission spectra were collected over the range of 300–550 nm with both excitation and emission slits set at 5 nm.

#### 2.3.5. Free Amino Content Determination

Free amino groups were measured to assess TGase-catalyzed covalent bond formation using an OPA assay [13]. Briefly, after 0.2 g samples were homogenized in 2 mL 0.1 M HCl, they were centrifuged at 10,000× *g* for 10 min. The supernatant (0.1 mL) was mixed with 2.5 mL fresh OPA reagent for 2 min. The absorbance of the mixture at 340 nm was measured.

#### 2.3.6. Confocal Laser Scanning Microscopy (CLSM)

CLSM was used to observe the microstructure of composite soymilk emulsion droplets. Specifically, 1 mL of the composite soymilk was stained with 40 μL of fluorescent dye solution. The fluorescent dye solution was prepared with 0.1% fluorescein isothiocyanate and 0.1% Nile Red. The stained samples were mounted on glass coverslips for microscopic examination.

### 2.4. Characterization of Composite Yuba Films

#### 2.4.1. Yield

Equation (1) was used to calculate the yield of the composite yuba:

(1) $$Yield(\%)=\frac{m}{100+X}\times 100$$

where *X* is the weight of the WPI (g), and *m* is the total weight of the dried yuba (g).

#### 2.4.2. Cooking Loss

The cooking loss was determined following the method described by Liu et al. [14].

#### 2.4.3. Mechanical Properties Ananlysis

The films formed in later stages exhibited low protein content during continuous yuba film production, which led to a decline in film quality [15,16]. Therefore, the first yuba film was chosen for further analysis. The yuba films were cut into strips of 2.0 cm × 7.0 cm based on the method reported by Kong et al. [17] before determining mechanical properties. The mechanical properties of yuba films were evaluated according to the method reported by Kong et al. [17]. A texture tester was used to determine the elongation at break (EAB) and tensile strength (TS). The testing parameters were as follows: the measurement speed = 1.0 mm/s, the pretest speed = 3.0 mm/s, and the post test speed = 5.0 mm/s. Software (Bluehill) was used to record the TS. Equation (2) was adopted to calculate the *EAB*:

(2) $$EAB(\%)=\left(L_{1}-L_{0}\right)/L_{0}\times 1$$

where *L*_0_ is the initial length of the yuba films (mm), and *L*_1_ is the length at break (mm).

#### 2.4.4. Fourier-Transform Infrared Spectrum (FTIR) Measurement

FTIR was performed according to the method reported by Yang et al. [18]. Potassium bromide was mixed with yuba powder at a mass ratio of 100:1 and thoroughly homogenized. Spectra were acquired over 64 scans in the 400–4000 cm^−1^ range at 4 cm^−1^ resolution. Peak Fit V 4.12 was adopted to analyze the secondary structures of the amide I region.

#### 2.4.5. X-Ray Diffraction (XRD)

XRD analysis was performed following a modified protocol based on Hu et al. [19]. The measurements were conducted with a scanning speed of 5°/min over a 2θ range of 5–90°. Other experimental parameters were as follows: tube current of 40 mA, tube voltage of 40 kV, and step size of 0.02°.

#### 2.4.6. SDS-PAGE

Protein samples (2 mg/mL) were mixed with loading buffer (4:1 volume ratio) and boiled for 5 min. After they were centrifuged at 6000 rpm for 5 min, the supernatant was loaded onto the gel to perform electrophoresis. The electrophoresis conditions were as follows: 80 V of stacking gel phase and 120 V of separating gel phase. Then, 0.1% Coomassie Brilliant Blue R-250 was used to stain the gel after electrophoresis for 30 min, and a mixture of 10% acetic acid and 50% methanol (*v*/*v*) was used to destain the gel. A BIO-RAD GelDocXR imaging system (Bio-Rad Laboratories, Inc., Hercules, CA, USA) was used to visualize the protein bands.

#### 2.4.7. Scanning Electron Microscopy (SEM)

A TM3030Plus scanning electron microscope (HITACHI, Tokyo, Japan) was used to observe the microstructures of the composite yuba. All the cut yuba films were fixed on a metallic table to be coated with a 5 nm layer of gold for imaging.

### 2.5. Statistical Data Analysis

The data are expressed as the mean ± standard deviation, and all the experiments were performed in triplicate. SPSS 26.0 was used to perform the statistical analysis. A TGase-based single-factor experimental design was adopted in this study. Data were subjected to one-way analysis of variance (ANOVA) followed by Duncan’s multiple test, with statistical significance set at *p* < 0.05.

## 3. Results and Discussion

### 3.1. Particle Size

**Figure 1:**
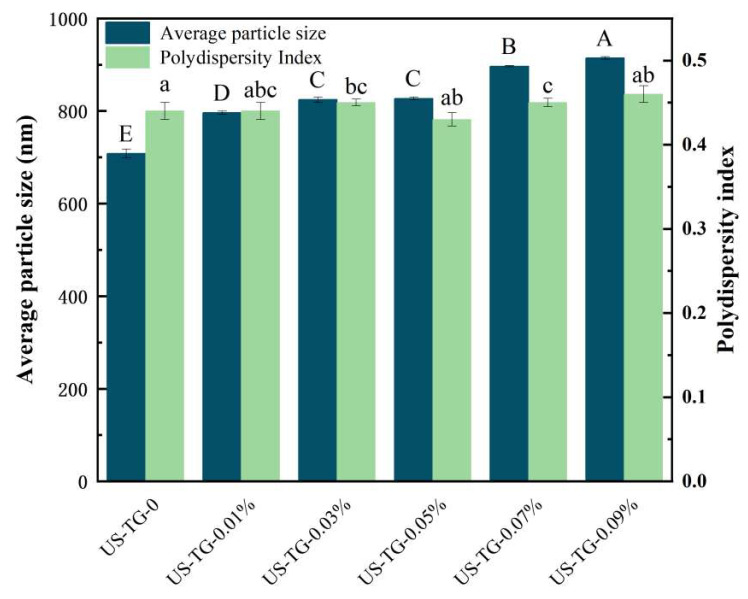
Effects of TGase concentration on particle size and PDI of composite soymilk. Different letters mean a significant difference between different groups at *p* < 0.05.

Effects of TGase concentration on the PDI and particle size of composite soymilk are shown in Figure 1. When the concentration of TGase rose from 0.01% to 0.09%, the particle size of composite soymilk significantly increased from 707.99 ± 9.47 nm to 914.41 ± 2.8 nm. The increase was attributed to the protein polymerization caused by TGase-catalyzed acyl transfer reactions [20]. The results were in accordance with the polymer formation shown in SDS-PAGE. Furthermore, when the concentration of TGase ranged from 0.01% to 0.05%, the PDI remained below 0.5, indicating that all the composite soymilk belonged to a relatively stable emulsion system. Previous studies suggested that proteins with an appropriate size could significantly enhance air–water interfacial properties by facilitating rapid migration, adsorption, and the formation of a viscoelastic interfacial layer. In contrast, excessively large protein aggregates may adversely affect interfacial behavior [6]. Therefore, the reduced yield observed in the US-TG-0.07% and US-TG-0.09% samples may be associated with their excessively large particle sizes.

### 3.2. SH Content

**Figure 2:**
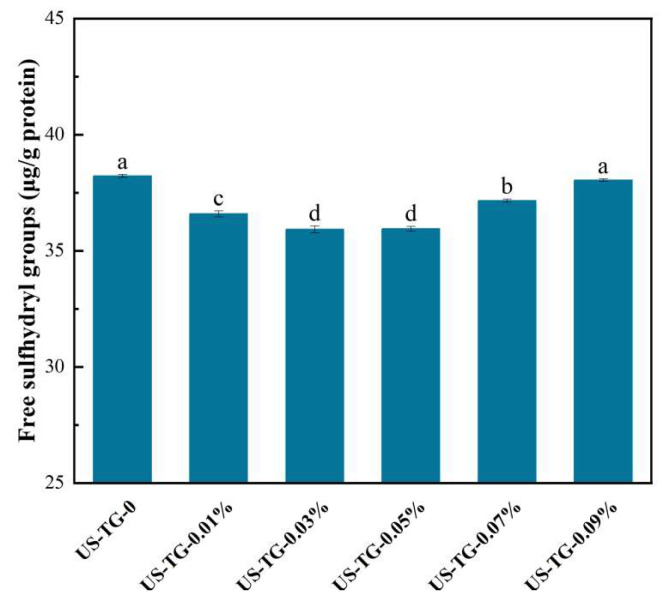
Effects of TGase concentration on free SH group contents of composite soymilk. Different letters mean a significant difference between different groups at *p* < 0.05.

SH groups are essential functional groups in protein structures, and their content directly reflects the cleavage and formation of disulfide bonds [18]. The reaction of sulfhydryl–disulfide (SH-SS) exchange plays a critical role in yuba formation [21]. Figure 2 shows the changes in the free SH content in composite soymilk with different concentrations of TGase. Adding TGase at concentrations between 0.01% and 0.07% significantly reduced the free sulfhydryl content, suggesting successful protein cross-linking. The SH content of composite soymilks with TGase concentrations of 0.03% and 0.05% was lower than that of the other composite soymilks. The reduction in SH suggested that the covalent cross-linking of composite soymilk protein was mediated by TGase. TGase could facilitate the enzyme-induced isopeptide cross-links and reduce intermolecular distances between proteins, which further promoted the contact between cysteine residues and formation of disulfide bonds [22]. These findings are in line with those of Chen et al. [23], who found that low concentrations of TGase decreased the free SH content in wheat flour. However, excessive TGase concentrations (>0.05%) accelerated protein cross-linking which further suppressed the cross-linking reactions and resulted in increased SH content [24].

### 3.3. Protein Tertiary Structure

**Figure 3:**
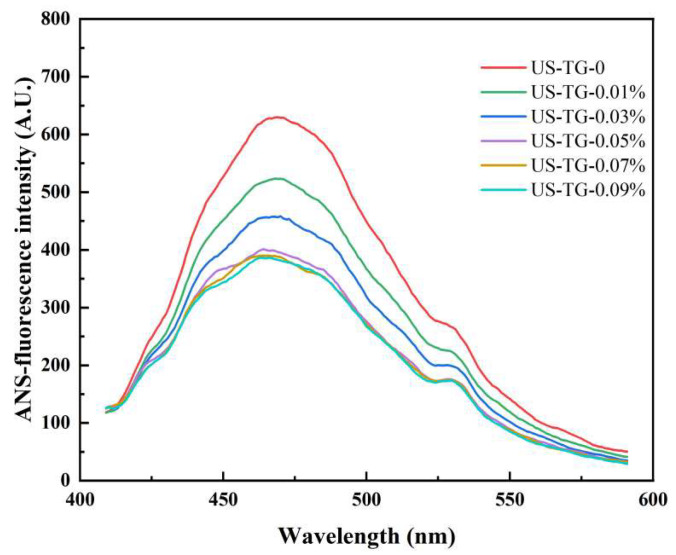
Effects of TGase concentration on surface hydrophobicity of composite soymilk.

The relative level of hydrophobic groups exposed on the protein surface could be reflected by protein surface hydrophobicity [25]. The surface hydrophobicity of composite soymilk significantly decreased when the TGase concentration rose (Figure 3). This could be related to TGase catalyzing the reactions of acyl transfer and facilitating isopeptide bond formation, which induced intermolecular and intramolecular cross-linking of proteins. This process buried hydrophobic groups, which further reduced hydrophobic site accessibility. The diminished accessibility of hydrophobic sites weakened their binding capacity with the hydrophobic probe of ANS [26]. Wang et al. [27] also reported that the cross-linking of mung bean protein isolate induced by TGase led to a decreased surface hydrophobicity.

**Figure 4:**
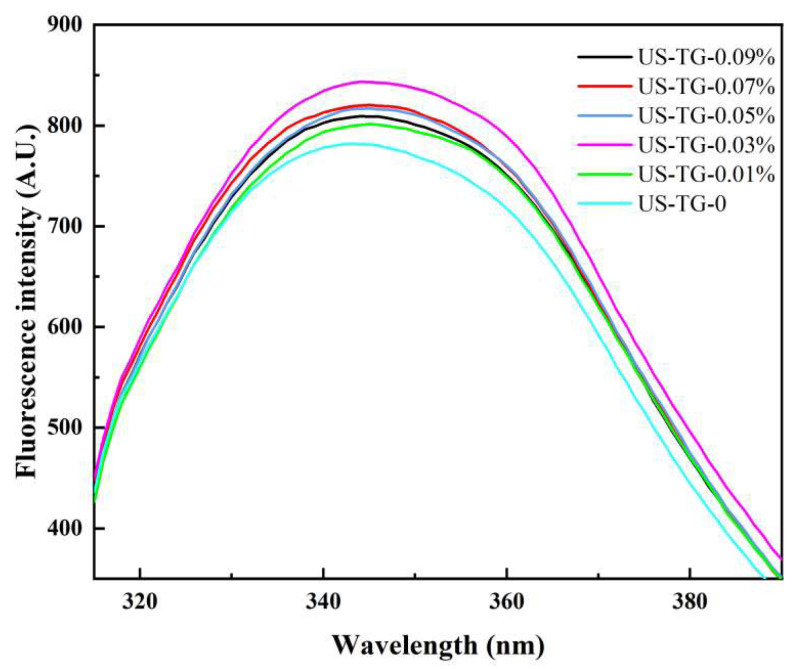
Effects of TGase concentration on intrinsic fluorescence of composite soymilk.

Fluorescence spectroscopy can track changes in protein tertiary structure [28]. The maximum fluorescence emission wavelength of composite soymilk with different TGase concentrations did not show a significant shift (Figure 4). The fluorescence intensity of composite soymilk rose with the increasing TGase concentration before the concentration of TGase reached 0.03%. This was likely due to protein aggregation exposing buried Tyr/Trp residues to the polar environment [6,22]. When the TGase concentration exceeded 0.03%, the fluorescence intensity began to decline. This indicated that TGase-induced polymerization exposed aromatic residues originally buried within the protein on the protein surface. The results regarding protein tertiary structure were in agreement with the result found by Li et al. [29].

### 3.4. Free Amino Content

**Figure 5:**
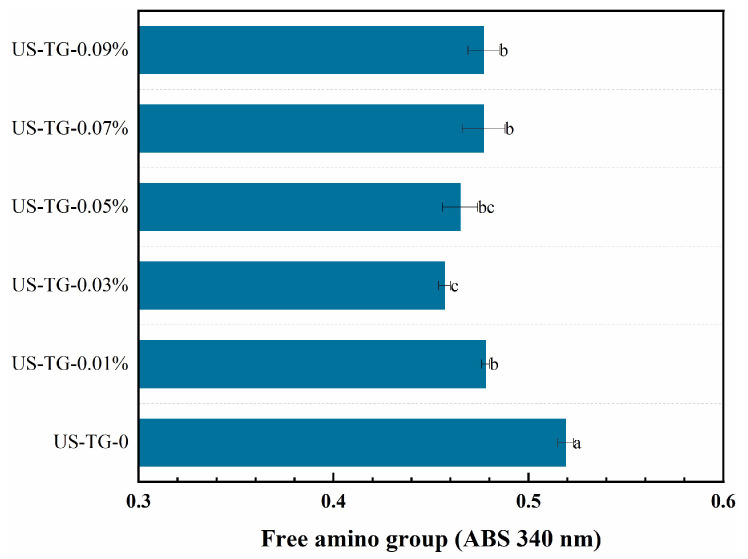
Effects of TGase concentration on free amino group contents of composite soymilk. Different letters mean a significant difference between different groups at *p* < 0.05.

A change in the free amino content could indicate TGase activity [30] due to the amino groups related to the cross-linking reaction. The free amino content of the TGase-treated composite soymilk is found to be significantly lower than that of the composite soymilk without TGase (*p* < 0.05) in Figure 5, indicating that TGase might induce protein–protein binding to generate γ-glutamyl lysine isopeptide bonds in composite soymilk, thereby reducing the free amino content [31]. Furthermore, Zhu et al. [32] found that these isopeptide bonds promoted protein folding and aggregation by linking helix structures together to form a more stable protein network. In addition, the composite soymilk treated with 0.03% TGase exhibited the highest cross-linking degree and the lowest content of free amino groups. These findings are consistent with those of Wen et al. [33], who observed that bitter apricot kernel protein with the addition of TGase had a lower free amino content than the protein without TGase. However, when TGase exceeded 0.03%, the free amino content of the composite soymilk did not decrease. When these residues are proximal in the protein structure, the cross-linking reaction of lysine and glutamine residues might become saturated [34]. Additionally, protein structure also influenced the extent of TGase-induced cross-linking.

### 3.5. CLSM

**Figure 6:**
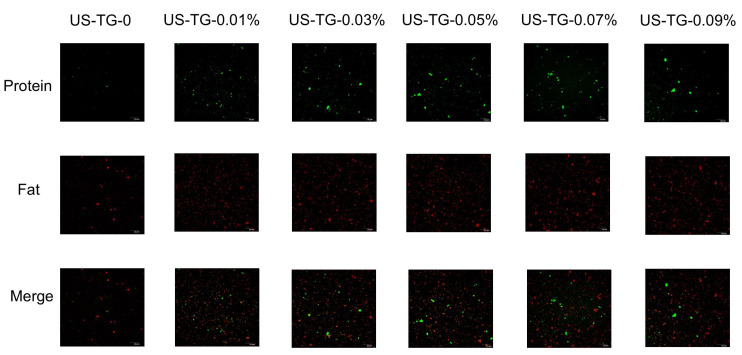
Confocal laser scanning microscopy results of composite soymilk treated with different TGase concentrations.

CLSM was employed to observe the microstructure of particles in composite soymilk with different concentrations of TGase. Figure 6 displays the morphology of TGase-treated composite soymilk and composite soymilk without TGase. The green fluorescent spots indicate the formation of protein aggregates, while the red fluorescent spots represent oil droplets, which distinguish the differences in oil droplet distribution and protein localization. Compared to the composite soymilk without TGase, the TGase-treated composite soymilk displayed smaller droplet sizes with a uniform distribution and exhibited larger green fluorescent spots, indicating that protein aggregates were formed. The varying sizes and irregular shapes of these aggregates were caused by protein cross-linking during the TGase-induced film formation process [35]. Limited cross-linking enhances coalescence stability, and extensive cross-linking might reduce the stability of protein-stabilized emulsions [36]. The droplet coalescence and protein aggregation induced by TGase treatment did not affect the stability of the composite soymilk.

### 3.6. Protein Secondary Structure

**Figure 7:**
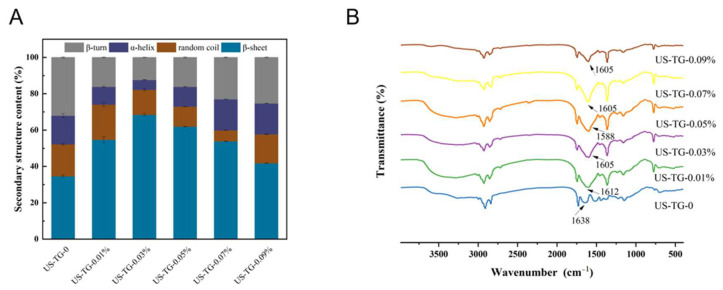
FTIR spectra of composite yuba treated with different TGase concentrations (**A**); effects of different TGase concentrations on secondary structure contents of composite yuba (**B**).

Figure 7A shows the FTIR spectra of the composite yuba with different concentrations of TGase. The band at 2925 cm^−1^ represents the C-H stretching vibration, and the band at 1526 cm^−1^ (amide II band) corresponds to the N-H bending vibration. The bands at 3000–3500 cm^−1^ are related to the O-H stretching vibration and N-H bending vibration. The peak at 1238 cm^−1^ (amide III band) is related to the C-N stretching vibration. The peak at 1625 cm^−1^ (amide I band) corresponds to the C=O stretching vibration [37]. The FTIR spectra of all the composite yuba did not show significant differences, indicating that the functional groups of the composite soymilk were not affected by the concentration of TGase. However, it is noteworthy that the amide I, amide II, and amide A bands of the composite yuba had higher absorption intensities than those of the composite yuba without TGase, which was related to the formation of isopeptide bonds [38].

The amide I band (1600–1700 cm^−1^, C=O stretching vibrations of protein backbone) in Figure 7B was subjected to deconvolution analysis to further analyze the changes in the protein secondary structure [39]. As shown in Figure 7B, when the concentration of TGase increased from 0% to 0.03%, the β-sheet content of the composite yuba significantly rose, while the α-helix content gradually decreased. This indicated a conversion from the α-helix structure of composite yuba to β-sheet, which might result from covalent cross-link formation between isopeptide bonds and disulfide bonds catalyzed by TGase, thereby promoting the generation of macromolecular protein polymers. The increased β-sheet content in the composite soymilk protein led to a more densely packed hydrogen bond network, which further enhanced both the overall structural stability and extensibility of the protein. The macromolecular polymers formed via TGase catalysis may further influence the stability of hydrogen bond breakage and reorganization in the composite protein. These changes ultimately led to structural alteration in composite yuba proteins [27].

### 3.7. XRD

**Figure 8:**
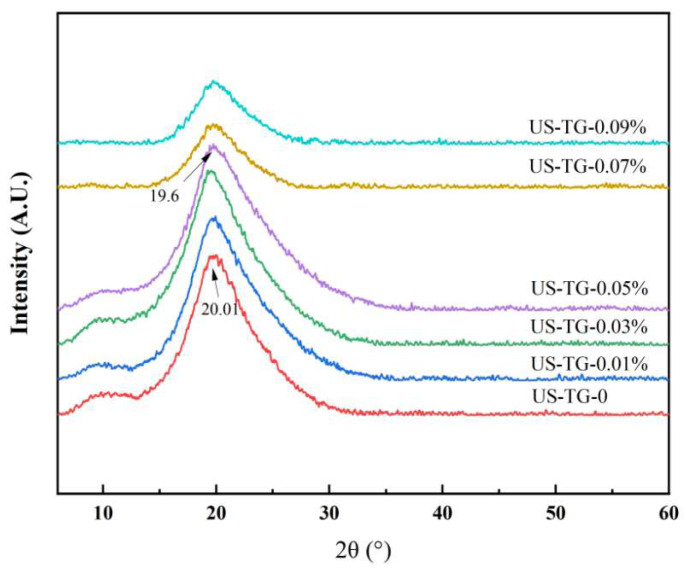
The XRD patterns of composite yuba treated with different concentrations of TGase.

XRD was employed to investigate the effect of different concentrations of TGase on the conformational changes in composite yuba. All yuba composites showed characteristic broad diffraction peaks centered at 2θ values of 9° and 20° (Figure 8), indicating that both soy protein and whey protein have amorphous characteristics. With the increasing concentration of TGase, the diffraction peak intensity showed an initial rise followed by a progressive decline. As more covalent cross-linking between the proteins of composite yuba appeared at lower TGase concentrations (<0.05%), rapid protein aggregation at higher TGase concentrations could lead to heterogeneous cross-linking [38]. Notably, the XRD peak positions of composite yuba were significantly modified by TGase treatment. TGase treatment induced significant alterations in the characteristic XRD peak positions of composite yuba. These changes likely resulted from structural rearrangements in β-sheets and α-helixes, thereby modifying the structure of the film matrix. With the increasing TGase concentration, the characteristic peak progressively shifted leftward from 20.01° to 19.6° (US-TG-0.05%), which might be related to the increment in β-sheet content [40]. The XRD results demonstrated that the rearrangement of protein secondary structures in the composite soymilk along with enhanced interactions between the WPI and soy proteins synergistically improved the structural density and modified the mechanical properties of composite yuba. These findings demonstrated that TGase treatment enhanced intermolecular interactions between soy protein and the WPI, promoting a denser structure.

### 3.8. SDS-PAGE Analysis

**Figure 9:**
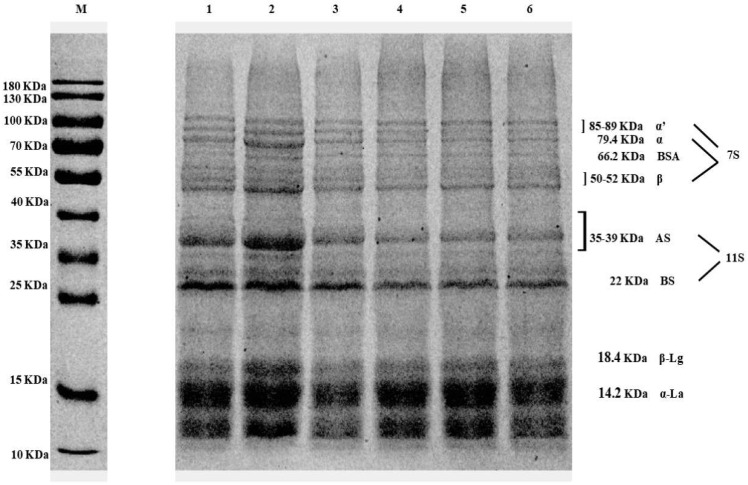
SDS-PAGE of composite yuba treated with different TGase concentrations. M: protein marker; 1: US−TG−0; 2: US−TG−0.01%; 3: US−TG−0.03%; 4: US−TG−0.05%; 5: US−TG−0.07%; 6: US−TG−0.09%.

SDS-PAGE analysis revealed changes in protein cross-linking induced by varying TGase levels in composite yuba. Figure 9 displays the distinct bands corresponding to soy protein and the WPI in all the samples. Additionally, the bands of the WPI and soy protein at 18.4~89 kDa became notably fainter with the increasing TGase concentration, compared to the composite yuba without TGase addition. The subunit structures at 39~89 kDa correspond to 7S and 11S (AS band), while those at 18.4 kDa correspond to β-lactoglobulin. The observed behavior likely resulted from TGase-mediated formation of covalent linkages resistant to SDS and mercaptoethanol cleavage during electrophoretic analysis [41]. These findings suggested that these protein subunits were the main substrates of TG in the composite yuba. The same phenomenon was also observed by Hui and Xing [42], who investigated the cross-linking of tofu following TG treatment.

### 3.9. Mechanical Properties

**Figure 10:**
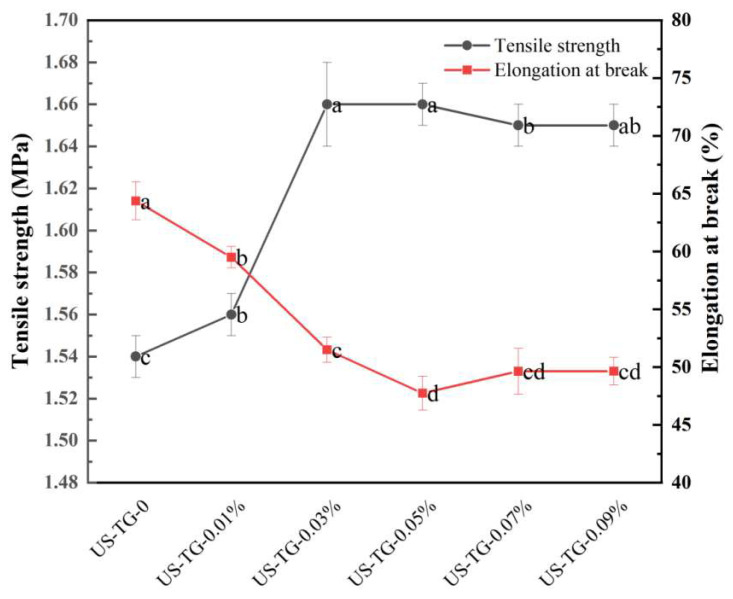
Effects of different TGase concentrations on mechanical properties of composite yuba. Different letters mean a significant difference between different groups at *p* < 0.05.

The network architecture of protein-based films governs their mechanical characteristics through the complex interplay of both inter- and intramolecular forces [43]. TGase significantly enhanced the TS of composite films (Figure 10). The TS value with 0–0.05% TGase ranged from 1.54 ± 0.01 MPa to 1.66 ± 0.02 MPa, and the TS value of composite films with 0.05% and 0.03% TGase was higher than that of other composite films. The TS values of composite films with 0.07% and 0.09% TGase showed no significant differences, suggesting the saturation of cross-linking reactions. This result was mainly due to the TGase-induced intramolecular polymerization of proteins improving the tensile strength [44]. In contrast, as the concentration of TGase increased from 0 to 0.09%, the EAB of WPI–composite yuba films decreased from 64.37 ± 1.64% to 49.65 ± 1.19%. The EAB of composite yuba with 0.05–0.09% TGase was lower than that of composite yuba with other addition levels of TGase. Kaewprachu et al. [45] also found that the EAB in fish myofibrillar protein films was reduced with increasing TGase levels. The reduction might result from TGase-mediated alterations enhancing structural rigidity at the expense of film elasticity [8]. However, some studies have reported opposing relationships between TGase and mechanical properties in protein-based films [46,47], which was attributed to the differential effects of proteins from different sources under TGase treatment. The mechanical performance of TGase-treated films positively correlated with the cross-linking degree, and higher cross-linking density yielded superior mechanical properties [48]. Therefore, suitable concentrations of TGase increased the TS value of the composite yuba and decreased its EAB value.

### 3.10. Yield and Cooking Loss

**Figure 11:**
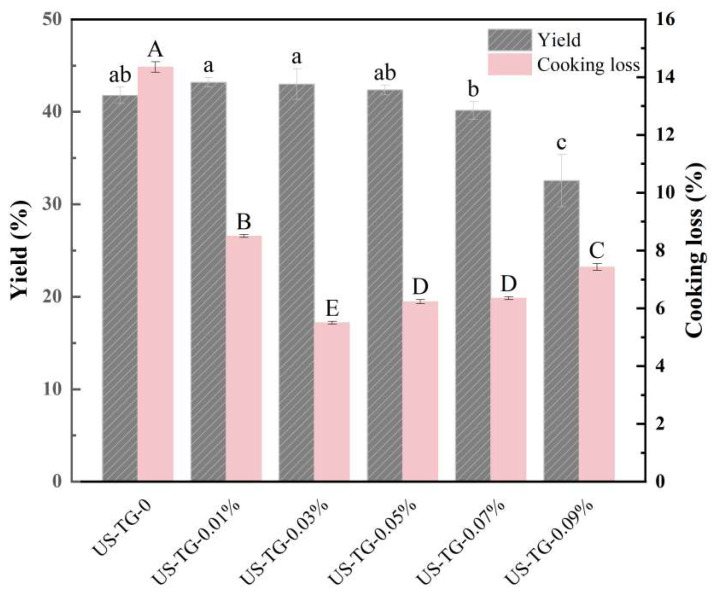
Effects of TGase concentration on the yield and cooking loss of composite yuba. Different letters mean a significant difference between different groups at *p* < 0.05.

The yield and cooking loss of composite yuba with different concentrations of TGase are displayed in Figure 11. The yield of composite yuba without TGase and with 0.01–0.05% levels of TGase showed no significant differences. However, the yield of composite yuba with a concentration higher than 0.05% began to decline. As high TGase concentrations induced excessive intermolecular cross-linking, oversized protein aggregates were formed, which aligns with the trend of particle size. The homogeneity of the protein network was disrupted by excessive cross-linking, which affected the formation efficiency and structural integrity of the yuba film and ultimately reduced the yuba yield.

As a key quality indicator of yuba, cooking loss primarily results from fat leaching and the dissolution of water-soluble proteins, which is related to the fat-binding capacity of yuba and the protein network structure. With the increasing concentration of TGase, the cooking loss of TGase-treated composite yuba significantly decreased (*p* < 0.05). The composite yuba with 0.03% TGase reached a minimum cooking loss of 5.5 ± 0.05%. The result suggests that TGase catalyzed the formation of a denser protein matrix in yuba through enhanced cross-linking, which effectively reduced the dry matter loss of yuba. However, when the TGase concentration exceeded 0.03%, the cooking loss showed an increasing trend. Feng et al. [49] also observed that Frankfurt sausages exhibited lower cooking loss at 0.1% and 0.3% TGase compared to sausages without TGase and showed higher cooking loss when the TGase concentration reached 0.5%.

### 3.11. SEM

**Figure 12:**
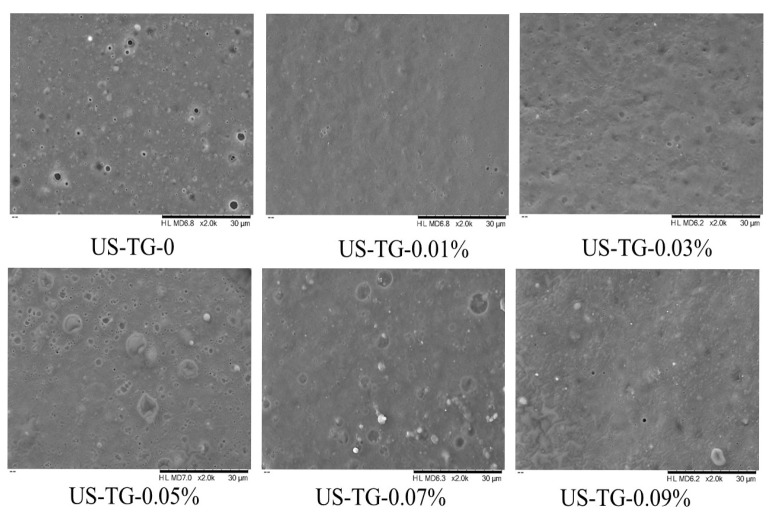
Microstructure of composite yuba treated with different TGase concentrations.

The microstructure of composite yuba treated with different TGase concentrations is shown in Figure 12. The SEM analysis of yuba revealed that composite yuba without TGase exhibited distinct micropores on its surface at high magnification. This structural characteristic appeared to result primarily from the formation of small cavities and cavitation microbubbles induced by ultrasonic pretreatment in the composite soymilk. During yuba film formation, the collapse of these cavitation bubbles under higher ultrasonic power generated high-speed liquid microjets and shock waves. Furthermore, prolonged water evaporation caused by continuous heating may also lead to distinct micropores. The surface pores in yuba could destroy its mechanical properties, as they represent weak points in the material that are prone to fracture and deformation under stress. Notably, TGase treatment eliminated these surface defects of composite yuba. The composite yuba with 0.01% TGase concentration displayed the smoothest surface morphology, which was likely due to low-concentration-TGase-induced covalent cross-linking enhancing the compatibility of the WPI and soy protein. However, the yuba surface gradually showed irregular protrusions and depressions as the TGase concentration increased. The structural deterioration might result from excessive protein cross-linking caused by high TGase levels, which compromised the uniformity and density of the protein network [50]. These observations were aligned with those of Jiang et al. [51], who found that whey protein concentrate–egg white protein composite films treated with TGase above 10 U/g protein exhibited protein aggregation and fibrous structures.

## 4. Conclusions

TGase modification could improve the film-forming capacity and physicochemical properties of ultrasound-treated composite soymilk. All the levels of TGase increased the particle size of the composite soymilk. The 0.01–0.07% addition level of TGase significantly decreased the SH and free amino group content of composite soymilk, suggesting that TGase facilitated the formation of disulfide bonds and isopeptide bonds. TGase addition significantly increased the tensile strength of composite yuba, which displayed the optimal mechanical performance of yuba. Moreover, TGase could reduce the cooking loss rate during industrial yuba production. With an increasing TGase concentration, the cooking loss of TGase-treated composite yuba showed a decreasing tendency. It is noteworthy that although the addition of TGase implies certain additional costs in the production of composite yuba, the quality of the yuba is markedly improved. Structural modification of composite soymilk proteins may occur through TGase-catalyzed acyl transfer, leading to improved physicochemical properties in the derived yuba products. However, the effects of TGase on the solubility, emulsifying properties, and nutritional value of the composite soymilk were not investigated in this study. TGase-cross-linked composite soymilk can also be applied in various fields including edible films, functional food ingredients, and 3D food printing materials in the future. These findings confirmed that the addition of TGase into composite soymilk at suitable levels serves as an effective cross-linking strategy for enhancing yuba quality.

## Data Availability

The original contributions presented in this study are included in the article/Supplementary Materials. Further inquiries can be directed to the corresponding author.

## Supplementary Materials

The following supporting information can be downloaded at: https://www.mdpi.com/article/10.3390/foods14162916/s1, Supplementary Materials picture.
