# Supplementary figures and images for: Characterization of Whey Protein Isolate–Soymilk Complexes Modified by Transglutaminase and Their Application inYuba Film
Source: Foods. 2025 Aug 21;14(16):2916. doi: 10.3390/foods14162916 (PMC12385331; doi:10.3390/foods14162916)

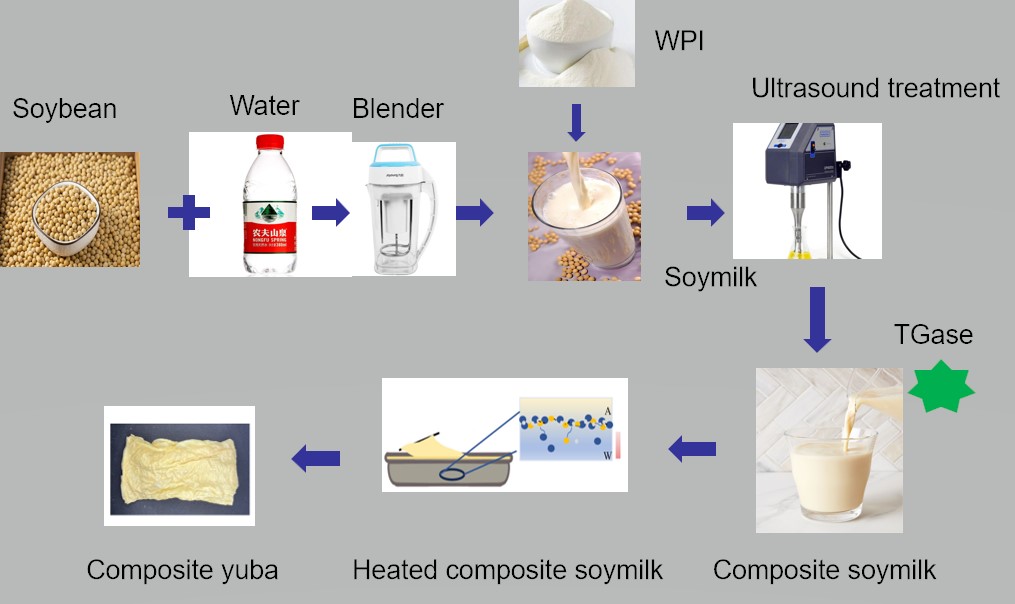

Supplement: Supplementary file 1 [file foods-14-02916-s001.zip › foods-3785639-supplementary.jpg]
